# Effect of B7.1 Costimulation on T-Cell Based Immunity against TAP-Negative Cancer Can Be Facilitated by TAP1 Expression

**DOI:** 10.1371/journal.pone.0006385

**Published:** 2009-07-24

**Authors:** Xiao-Lin Li, Yong-Yu Liu, David Knight, Yoshinobu Odaka, J. Michael Mathis, Runhua Shi, Jonathan Glass, Qian-Jin Zhang

**Affiliations:** 1 Department of Cellular Biology and Anatomy, Gene Therapy Program, Feist-Weiller Cancer Center, Louisiana State University Health Sciences Center, Shreveport, Louisiana, United States of America; 2 Feist-Weiller Cancer Center, Louisiana State University Health Sciences Center, Shreveport, Louisiana, United States of America; 3 College of Pharmacy, Basic Pharmaceutical Sciences, University of Louisiana, Monroe, Louisiana, United States of America; New York University School of Medicine, United States of America

## Abstract

Tumors deficient in expression of the transporter associated with antigen processing (TAP) usually fail to induce T-cell-mediated immunity and are resistant to T-cell lysis. However, we have found that introduction of the B7.1 gene into TAP-negative (TAP^−^) or TAP1-transfected (TAP1^+^) murine lung carcinoma CMT.64 cells can augment the capacity of the cells to induce a protective immune response against wild-type tumor cells. Differences in the strength of the protective immune responses were observed between TAP^−^ and TAP1^+^ B7.1 expressing CMT.64 cells depending on the doses of γ-irradiated cell immunization. While mice immunized with either high or low dose of B7.1-expressing TAP1^+^ cells rejected TAP^−^ tumors, only high dose immunization with B7.1-expressing TAP^−^ cells resulted in tumor rejection. The induced protective immunity was T-cell dependent as demonstrated by dramatically reduced antitumor immunity in mice depleted of CD8 or CD4 cells. Augmentation of T-cell mediated immune response against TAP^−^ tumor cells was also observed in a virally infected tumor cell system. When mice were immunized with a high dose of γ-irradiated CMT.64 cells infected with vaccinia viruses carrying B7.1 and/or TAP1 genes, we found that the cells co-expressing B7.1 and TAP1, but not those expressing B7.1 alone, induced protective immunity against CMT.64 cells. In addition, inoculation with live tumor cells transfected with several different gene(s) revealed that only B7.1- and TAP1-coexpressing tumor cells significantly decreased tumorigenicity. These results indicate that B7.1-provoked antitumor immunity against TAP^−^ cancer is facilitated by TAP1-expression, and thus both genes should be considered for cancer therapy in the future.

## Introduction

One effective strategy against cancer is the induction of CD8^+^ T-cell immunity [1]. However, tumors deficient in major histocompatibility complex class I (MHC-I) antigen presentation often evade immune-mediated destruction [2], [3], . Deficiency in TAP expression is frequently observed in human cancer [2], [3]. TAP is composed of TAP1 and TAP2 subunits whose function is to transport cytosolic-generated peptides into the lumen of the endoplasmic reticulum (ER) for MHC-I binding, followed by transport of MHC-I/peptide complexes to the cell surface. Lack of TAP expression (one or both subunits) in tumor cells generally inhibits expression of TAP-dependent peptide antigens on the cell surface, resulting in failure of CD8^+^ T-cell recognition.

Although presentation of TAP-dependent peptide antigens is limited, TAP-deficient cells are able to present TAP-independent antigens. For example, mouse T-lymphoma RMA-S cells which express only TAP1 [6] and human T and B hybrid T2 cells which lack both TAP1 and TAP2 genes [7] can present some MHC-I restricted TAP-independent antigens derived from proteins located in or outside the ER lumen [8], [9], [10], [11], [12], [13]. Different capacities for antigen presentation are also observed between TAP-negative and TAP1-positive tumor cells. Gabathuler, R et al. report that introduction of the TAP1 gene into TAP-negative tumor cells changes the mode of antigen presentation to resemble that of TAP1-positive RMA-S cells, as indicated by the fact that a virally-derived antigen can be efficiently presented on the surface of TAP1-possitive but not TAP-negative tumor cells [14]. Even though TAP-independent antigens can be presented by TAP1-positive or TAP-negative tumor cells, failure of the cells to efficiently induce a large population of tumor-antigen specific CD8^+^ T-cell effectors may be a major reason limiting efficacy of antitumor immunity. Such antitumor immunity can be augmented by introducing a B7.1 gene into TAP-deficient tumor cells as reports indicated [13], [15].

B7.1 is a costimulatory molecule that interacts with CD28 on T lymphocytes and plays an important role in immune induction. Tumors transduced with B7.1 can elicit CD8^+^ T-cell dependent antitumor responses [16], [17], [18]. In particular, B7.1 transfected TAP1-expressing RMA-S cells can elicit T-cell clones reacting with TAP1 or TAP2 deficient tumor cells, but not with TAP-competent tumor cells [13]. One such T-cell clone recognizes an ER-localized-Lass5-protein-derived antigen that is presented exclusively by RMA-S and other TAP1 or TAP2 deficient cells and protects mice from RMA-S tumor challenge [13]. These studies provide useful information regarding the priming of T-cell based immunity against tumor immune escape variants in cancer immunotherapy. However, in many cases, human cancer exhibits a TAP-negative phenotype, and thus it is still unclear whether B7.1 expression in these tumor cells can elicit the protective T-cell immunity or whether expression of both B7.1 and TAP1 genes is required to generate such immunity.

In this study, we used a murine lung carcinoma CMT.64 cell line as a model cell system to address this question. The CMT.64 cells are defective in both TAP1 and TAP2 at transcriptional and translational levels and express low levels of MHC-I molecules [14], [19]. We tranfected the CMT.64 cells with either B7.1 gene alone or B7.1 together with mouse TAP1 genes, and found that transfectants induced different antitumor immune responses. B7.1 and TAP1 co-expression in tumor cells significantly decreases their tumorigenicity while B7.1 expression alone in the cells has no change. However, both B7.1-expressing and B7.1 and TAP1 co-expressing cells dramatically increased the capacity for generation of protective immunities at the high dose of tumor immunization. At the low dose of tumor immunization, only B7.1 and TAP1 co-expressing cells provided protective immunity. These results suggest that B7.1 and TAP1 co-expression in tumor cells is important for T-cell priming.

## Results

### B7.1 and TAP1 expression decreases tumorigenicity of TAP-deficient CMT.64 cells only in T-cell competent mice

**Figure 1:**
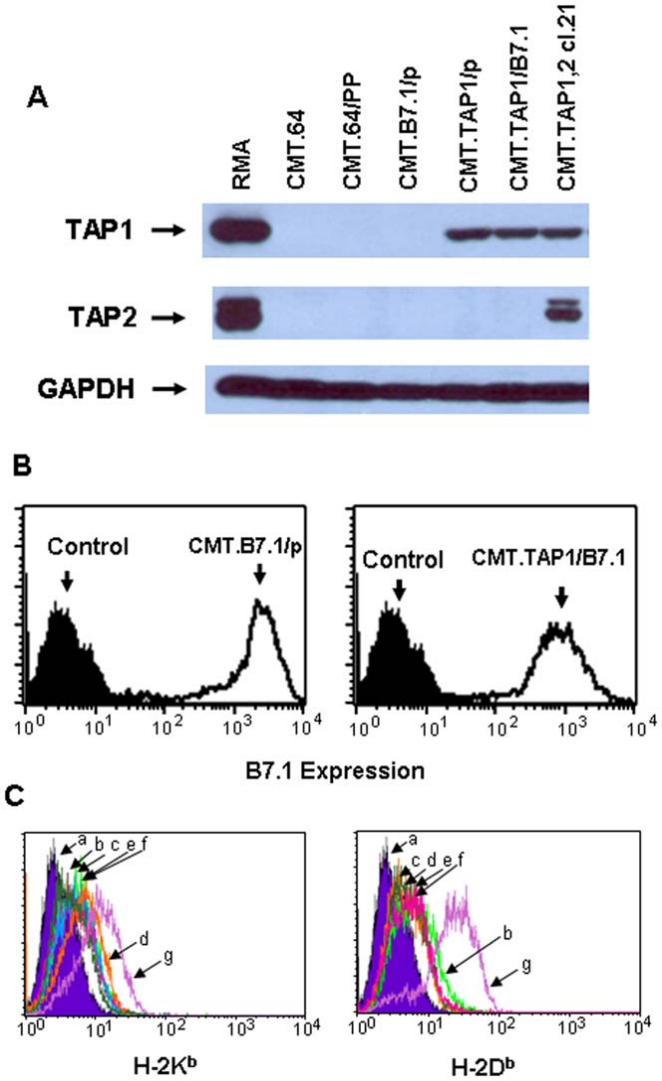
Expression of TAP1, B7.1 and MHC-I molecules in transfectants. TAP, B7.1, K^b^ and D^b^ expression in CMT.64 transfectants was determined. A) TAP1 and TAP2 proteins were detected in CMT.64 transfectants and control RMA cells by Western blots using anti-TAP1 and TAP2 polyclonal antibodies respectively (see Material and Methods). Levels of expression of GAPDH protein were detected in each sample as the loading control. B) B7.1 expression in CMT.B7.1/p and CMT.TAP1/B7.1 cells was detected by FACS assays using FITC-conjugated B7.1-specific mAb 16-10A1. Control is CMT.64 cells stained with mAb 16-10A1. C) K^b^ or D^b^ expression was detected by FACS assays using primary mAbs Y-3 against H-2K^b^ or 28-14-8S against H-2D^b^ followed by staining with a FITC-conjugated goat anti-mouse IgG secondary Ab. CMT.64 cells stained with a primary mAb 15-5-5S (against H-2D^k^) followed by staining with a FITC-conjugated goat anti-mouse IgG secondary Ab were used as a negative control. a: negative control; b: CMT.64; c: CMT.64/pp; d: CMT.B7.1/p; e: CMT.TAP1/p; f: CMT.TAP1/B7.1; and g: CMT.TAP1,2 cl.21.

To evaluate the effects of B7.1 expression on TAP1-positive and TAP-negative tumor cells on antitumor immunity, we first analyzed expression of the introduced genes in CMT.64 transfectants. CMT.64/pp, CMT.B7.1/p, CMT.TAP1/p, CMT.TAP1/B7.1 and CMT.TAP1,2 cl.21 cells were transfected with two empty vectors, B7.1+empty vector, TAP1+empty vector, TAP1+B7.1 or TAP1+TAP2, respectively (see Material and Methods for detail). After drug selection, the transfectants expressed the relevant gene(s), except for a CMT.64/pp transfectant which did not show TAP1, TAP2 or B7.1 expression (Fig.1A and 1B). Gene expression was stable during the time of study. K^b^ and D^b^ expression in most transfectants was similar to wild-type CMT.64 cells, except for CMT.TAP1,2 cl.21 cells which expressed K^b^ and D^b^ molecules at levels higher than that expressed in the others (Fig. 1C).

**Figure 2:**
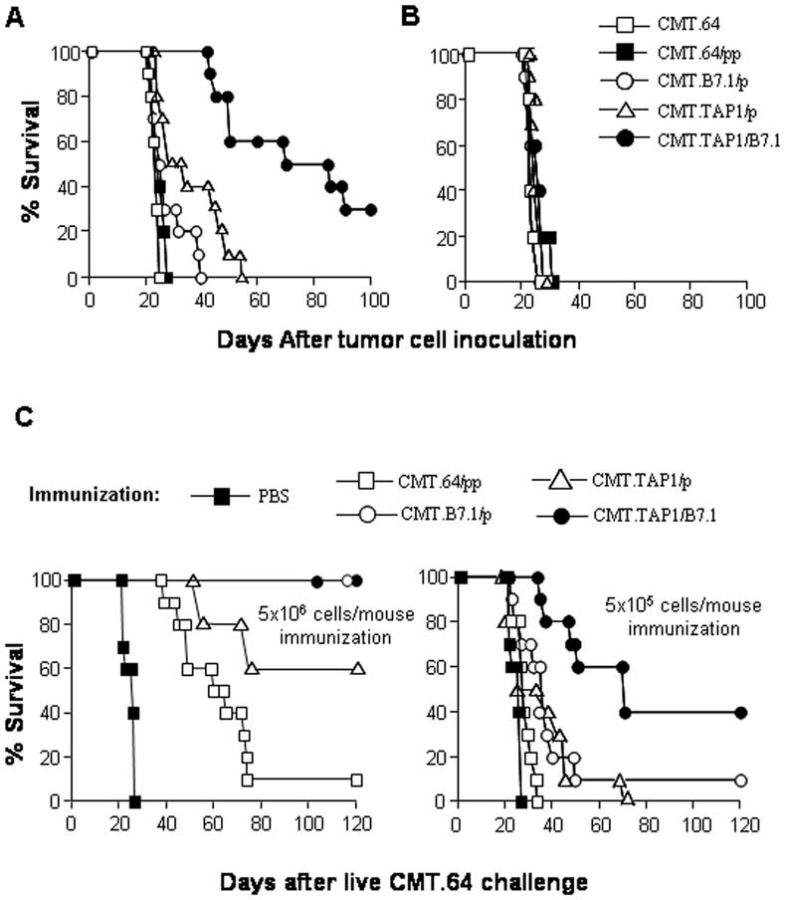
Decrease in tumorigenicity and increase in immune response by B7.1 and TAP1 co-expressing tumor cells. The time of morbidity was recorded for mice (each group, n = 10) inoculated with live tumor cells or immunized with γ-irradiated cells and followed by challenge with CMT.64 cells. Statistics for mouse survival were obtained using the Kaplan–Meier log rank survival test and differences were considered significant at P<0.05. A) Tumorigenicity was detected by injection of C57BL/6 mice i.p. with live CMT.64 cells or their transfectants (5×10^4^ cells per mouse). B) Nude mice were used for determination of tumorigenicity with conditions of tumor injection the same as shown in A), P>0.05 for all comparisons. C) C57BL/6 mice were immunized i.p. with different γ-irradiated tumor cells or PBS at a high-dose (left, 5×10^6^ cells per mouse) or a low-dose (right, 5×10^5^ cells per mouse). After a 20-day immunization, the mice were challenged i.p. with live CMT.64 tumor cells (2.5×10^5^ cells per mouse). Different survival rates and/or times were observed.

To assess whether B7.1 expression in the tumor cells could decrease tumorigenicity, C57BL/6 mice were injected with live CMT.64 cells or transfectants, and survival rates and/or times were recorded. Results are shown in Fig. 2A. All mice injected i.p. with CMT.64 or CMT.64/pp cells (2 control groups) died before day 27 after tumor cell inoculation. In contrast with two controls, CMT.TAP1/B7.1-bearing mice showed a highly significant survival rate (P≪0.05), even compared with all other mouse groups (P≪0.05). At day 100, 30% of the mice were still alive. However, CMT.B7.1/p-bearing mice showed no significant difference, compared to two control groups (P>0.05). CMT.TAP1/p-bearing mice showed significant survival time compared to the two controls (P<0.05) but no difference from the CMT.B7.1/p-bearing mice (P>0.05). Results of this experiment demonstrate that a substantial decrease in tumorigenicity is mediated by expression of B7.1 together with TAP1 molecules in CMT.64 tumor cells.

To determine if decreased tumorigenicity of CMT.TAP1/B7.1 cells was mediated by T cells, nude mice were inoculated with CMT.64 cells or transfectants, and survival was determined. Figure 2B shows that all mice died at 25–27 days, suggesting that T cells played an essential role in the immune response against CMT.TAP1/B7.1 cells, resulting in a decrease in tumorigenicity.

### CMT.B7.1/p-immunization at a high dose induces an immune response as efficient as CMTTAP1/B7.1-immunization but is less efficient at a low dose

Antitumor immune responses can be augmented by B7.1-expressing tumor cell immunization [16], [17], [18]. To assess whether protective immunity against TAP-negative tumor cells can be generated by immunization with either TAP1-expressing CMT.TAP1/B7.1 cells or TAP-negative CMT.B7.1/p cells, C57BL/6 mice were immunized with different amounts of γ-irradiated cells followed by challenge with CMT.64 cells. Control mice were immunized with γ-irradiated CMT.64/pp cells. Results indicate a dose-dependent protection. At the high dose of immunization (5×10^6^ cells per mouse), both CMT.TAP1/B7.1-immunized and CMT.B7.1/p-immunized mice were well protected from live tumor attack (Fig. 2C left-panel). The two mouse groups showed a highly significant survival rate (100% survivors), compared with the CMT.TAP1/p-immunized mouse group (60% survivors, p<0.05). The latter group showed a survival rate significantly higher than a control group immunized with TAP-negative CMT.64/pp cells (P≪0.05). However, at the low dose of immunization (5×10^5^ cells per mouse), CMT.TAP1/B7.1-immunized mice had a survival rate higher than CMT.B7.1/p-immunized mice (Fig. 2C right-panel, P<0.05). No difference in survival rate was observed between CMT.B7.1/p-immunized and CMT.TAP1/p-immunized mice (P>0.05). Results suggest that at the high dose of immunization, B7.1 and TAP1 co-expression or B7.1 expression alone renders tumor cells potent immunogens, while at low dose immunization, potent immunogenicity requires tumor cells co-expressing both TAP1 and B7.1 molecules.

### CD8^+^ T cells play an important role in mouse protection

**Figure 3:**
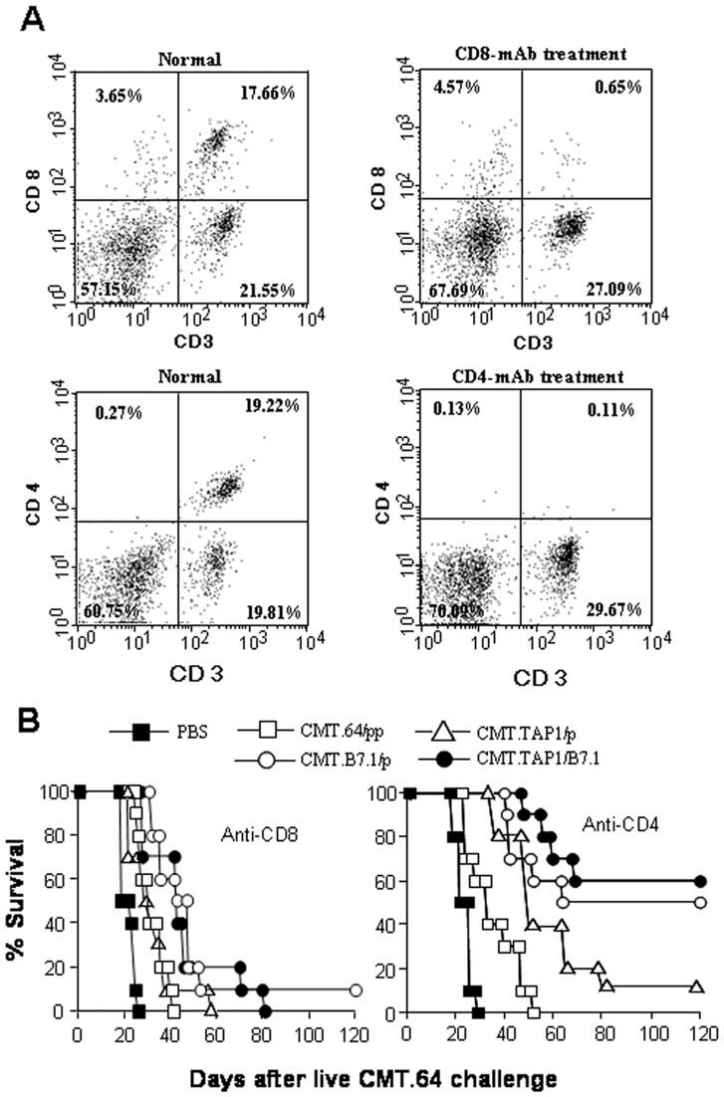
Deduction or abolishment of immune response against CMT.64 tumor by depleting CD4^+^ or CD8^+^ T cell sub-population in C57BL/6 mice. Mice (each group, n = 10) were injected i.p. with mAbs GK1.5 for CD4^+^ T cells or 2.43 for CD8^+^ T cells (0.1 ml per mouse) every other day for the first week and once per week afterward to deplete relevant T-cell sub-population. A) Before γ-irradiated tumor cell immunization, depletion of CD8^+^ or CD4^+^ T cell subsets was assessed in blood by FACS assays using FITC-conjugated anti-mouse CD8a (5H1-1) or FITC-conjugated anti-mouse CD4 (RM4-4) together with PE/Cy5-conjugated anti-mouse CD3 (145-2C11). Frequencies of CD8^+^ or CD4^+^ T cells were detected before mAb treatment (indicated as normal) and after first week mAb treatment and before γ-irradiated tumor cell immunization (indicated as mAb-treatment). B) After a 20-day immunization with γ-irradiated tumor cells (5×10^6^ cells per mouse) the mice were challenged i.p. with live CMT.64 tumor cells (2.5×10^5^ cells per mouse). The time of morbidity was recorded.

To determine if the increased protection after B7.1-expressing tumor immunization was mediated by a T-cell based immune response, we used mAbs to deplete CD8^+^ or CD4^+^ T cells in C57BL/6 mice before immunization and tumor cell challenge. Mice injected i.p. with relevant mAb every other day for the first week were analyzed by FACS assays for CD8^+^ or CD4^+^ T-cell population in blood. Results showed that the treatment dramatically decreased the CD8^+^ T-cell or CD4^+^ T-cell population (Fig. 3A). Anti-CD8 specific mAb treatment reduced CD8^+^ T cells from 17.66% to 0.65%, and anti-CD4 specific mAb treatment reduced CD4^+^ T cells from 19.22% to 0.11%. T-cell subpopulation depleted mice were injected with γ-irradiated CMT.64 transfectants, followed by challenge with live CMT.64 cells. Survival results are shown in Fig. 3B. The anti-CD8 specific mAb treatment significantly decreased the survival of tumor-bearing mice. There was no biologically significant difference between each tested-group and the control group (CMT.64/pp-immunized mice). In addition, the anti-CD4 specific mAb treatment partially decreased survival of each tested-group, compared with the control group (P≪0.05). Results suggest that protection of mice from tumor challenge fully depended on CD8^+^ T cells and partially depended on CD4^+^ T cells.

### Presentation of TAP-independent Lass5 antigen by TAP-deficient tumor cells

**Figure 4:**
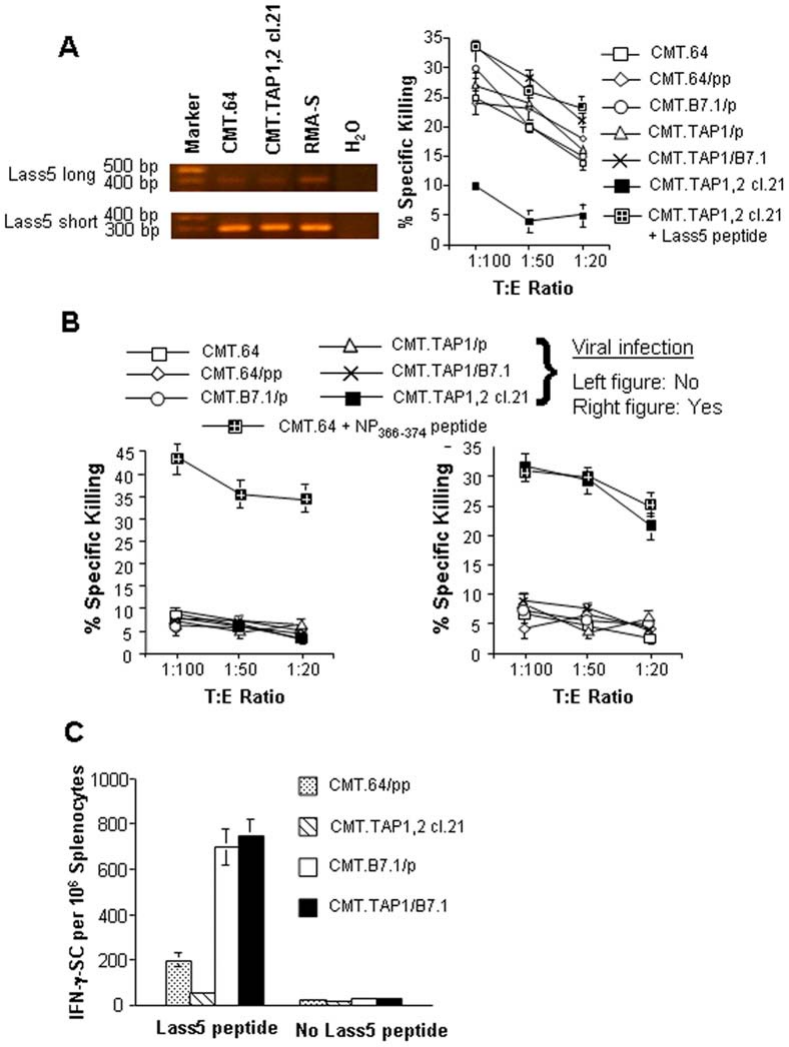
TAP-deficient tumor cells present TAP-independent antigen for T cell priming. A) Left: RT-PCR was performed to detect two spliced (long and short) Lass5 gene transcripts [13] in CMT.64, CMT.TAP1,2, cl.2 and RMA-S cells. Only the long transcript encodes a Lass5 epitope [13]. A) Right: Lass5 epitope presentation by TAP-deficient and TAP-proficient CMT.64 cells was detected by 12–16 h ^51^Cr-release assays. Lass5 specific T cells were generated by immunization i.p with γ-irradiated RMA-S/B7.1 cells pulsed with 5 µM Lass5 peptide. The immunized splenocytes were re-stimulated *in vitro* with Lass5 peptide-pulsed γ-irradiated RMA-S/B7.1 cells for 5 days. One of three experiments is shown. B) Left: 12–16 h ^51^Cr-release assays were conducted to confirm that an NP_366–374_ eptitope specific T-cell population generated by immunization with γ-irradiated RMA-S/B7.1 cells pulsed with 5 µM NP_366–374_ peptide did not contain T-cell sub-populations recognizing TAP-independent epitopes presented by CMT.64 and its transfectants. The ^51^Cr-labled targets were shown in figure 4B. B) Right: Standard ^51^Cr-release assays were conducted to confirm that TAP-deficient CMT.64 transfectants did not present TAP-dependent NP_366–374_ epitope when the cells were infected overnight with VV carrying NP_366–374_ minigene at multiplicity of infection (MOI) of 3. The ^51^Cr-labled targets were shown in figure 4B. The NP_366–374_ epitope specific T-cells were generated by immunization with γ-irradiated RMA-S/B7.1 cells pulsed with 5 µM NP_366–374_ peptide and re-stimulated with 5 µM NP_366–374_ peptide in vitro. C) An ELISPOT assay was performed to detect Lass5 specific IFN-γ-secreting precursors. Mice were immunized with γ-irradiated CMT.64/pp, CMT.TAP1,2, CMT.B7.1/p and CMT.TAP1/B7.1 tumor cells. The immunized splenocytes were stimulated with or without Lass5 peptide. The number of Lass5 antigen-specific, IFN-γ-secreting precursors was determined. Precursor frequency is reported as IFN-γ-secreting cells per 10^6^ splenocytes (IFN-γ-SC/10^6^ splenocytes).

The fact that TAP-deficient B7.1-expressing tumor cells can generate a CD8^+^ T-cell mediated immune response against TAP-negative tumor cells suggests that the tumor cells present TAP-independent antigen(s) for T-cell priming. To determine if this is true, we focused on the D^b^-restricted Lass5 epitope MCLRMTAVM, an antigen presented by many TAP-deficient cells [13]. CMT.64 and CMT.TAP1,2 cl.21 cells express the Lass5 gene as indicated by real-time PCR (Fig. 4A left). However, TAP-negative and TAP1-transfected CMT.64 cells but not TAP-competent CMT.TAP1,2 cl.21 cells were killed by a T-cell population generated by immunization with Lass5 peptide pulsed RMA-S/B7.1 cells as determined by a prolonged (12–16 h) ^51^Cr-release assay (Fig. 4A right). The TAP-competent CMT.TAP1,2 cl.21 cells were killed only when they were pulsed with Lass5 peptide (Fig. 4A right), suggesting that the cells did not efficiently present this epitope.

Since the T-cell population was generated by RMA-S/B7.1 cells pulsed with Lass5 peptide, we cannot exclude the possibility that the T-cell population contains sub-populations of other epitope-specific T cells that kill TAP-deficient tumor cells. This is because the RMA-S/B7.1 cells used for immunization have been reported to be able to generate many different K^b^ and D^b^ restricted T-cell clones [13]. To eliminate this possibility, we generated a D^b^-restricted influenza NP_366–374_ (ASNENMDTM) epitope specific T-cell population by immunization with RMA-S/B7.1 cells pulsed with NP_366–374_ peptide using conditions similar to those for Lass5 specific T-cell generation. Tumor cells do not express influenza NP_366–374_ epitope and thus NP_366–374_ epitope specific T cells should not recognize the tumor cells. If the T-cell population contains NP_366–374_ epitope irrelevant T cells, such a T-cell population can either kill TAP-deficient tumor cells or fail to kill the cells. If the T-cells kill the tumor cells this suggests that TAP-independent epitope specific T-cells, other than NP_366–374_ epitope, can be co-generated by immunization with RMA-S/B7.1 cells pulsed with the NP_366–374_ peptide. If the T-cells do not kill the tumor cells this suggests that the peptide-pulsed RMA-S/B7.1 cells generate a T-cell population containing T-cells that preferentially recognize the peptide epitope used for immunization. Results show that the generated T cells cannot kill either TAP-deficient or TAP-competent tumor cells except for CMT.64 cells pulsed with NP_366–374_ peptide (Fig. 4B left). Thus, our results confirm that TAP-deficient tumor cells present Lass5 epitope for T-cell recognition. In contrast with TAP-independent Lass5 epitope presentation, TAP-deficient tumor cells were unable to present the TAP-dependent and D^b^-restricted NP_366–374_ epitope when cells were infected with VV carrying the NP_366–374_ minigene (Fig.4 B right).

To determine if CMT.TAP1/B7.1, CMT.B7.1/p, CMT.TAP1/p and CMT.64/pp cells are able to elicit Lass5 specific T cells, the cells were injected i.p into C57BL/6 mice, and IFN-γ secreting splenocytes were quantified using an ELISPOT assay. As shown in Fig. 4C, a large increase in the number of Lass5 specific IFN-γ secreting splenocytes was observed in mice immunized with either CMT.TAP1/B7.1 or CMT.B7.1/p cells compared with mice immunized with CMT.TAP1/p or CMT.64/pp cells. These results indicate that TAP-negative and TAP1-expressing tumor cells can present TAP-independent antigens, including Lass5 antigen and that B7.1 expression in the tumor cells provides better T-cell priming.

### Immunization with CMT.64 cells infected with both VV-B7.1 and VV-TAP1 increases protection of mice from tumor challenge

**Figure 5:**
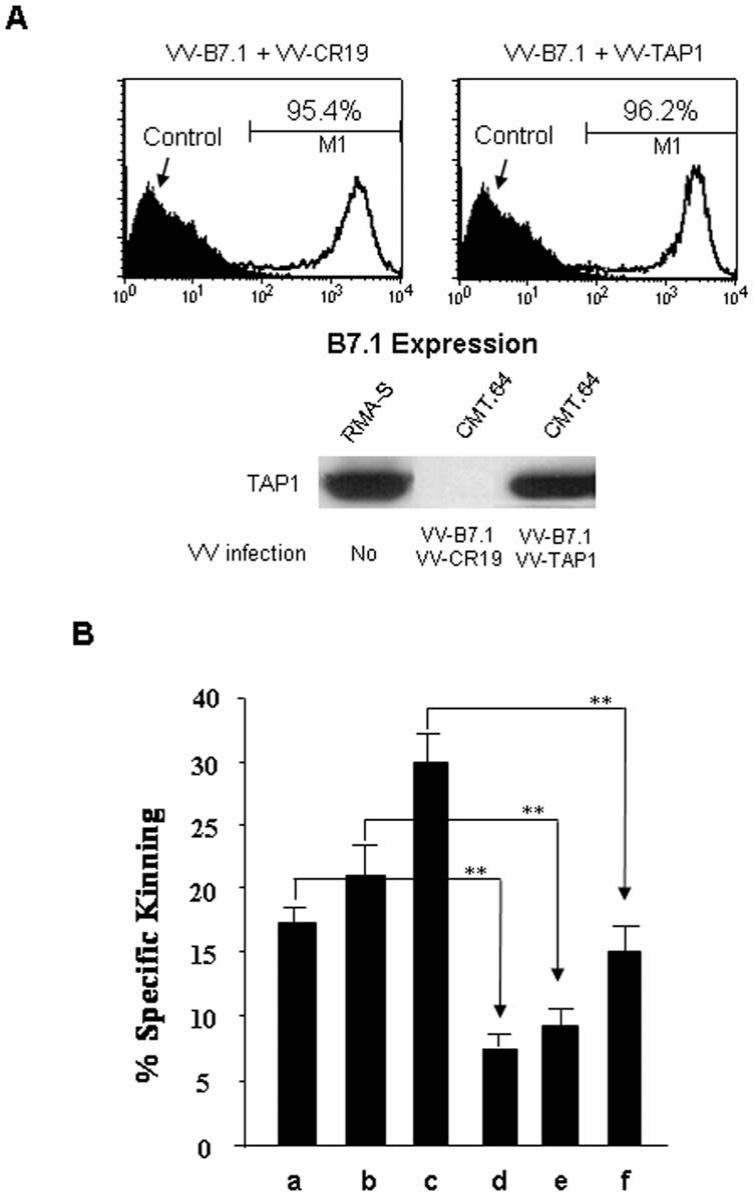
Influence of TAP-independent tumor antigen presentation in virally infected TAP-deficient tumor cells. CMT.64 cells were infected overnight with a combination of two VVs at MOI of 3 for each VV. A) B7.1 expression was detected by FACS assay using FITC-conjugated B7.1-specific mAb 16-10A1. CMT.64 cells without viral infection were used as a negative control. TAP1 expression was detected by Western blot using a goat anti-mouse TAP1 polyclonal Ab. RMA-S cells were used as a control. B) Standard ^51^Cr-release assays were conducted to detect if virally infected tumor cells affected presentation of endogenous tumor antigens. CMT.64 tumor cells were infected overnight with VV-GFP+VV-GFP, VV-B7.1+VV-GFP, or VV-B7.1+VV-TAP1, and used as target cells. Control cell lines were CMT.64, CMT.B7.1/p and CMT.TAP1/B7.1. Tumor antigen-specific T cells were generated by immunization with γ-irradiated CMT.B7.1/p cells. Target and effector ratio was used at 1∶100. a: CMT.64; b: CMT.B7.1/p; c: CMT.TAP1/B7.1; d: CMT.64+VV-GFP+VV-GFP; e: CMT.64+VV-B7.1+VV-GFP and f: CMT.64+VV-B7.1+VV-TAP1. ** indicates statistical significance (P≪0.05) using ANOVA analysis.

A potential method for more reliable cancer immunotherapy is use of a viral-based gene-carrying vector to infect cells for immunization. Since immunization with a high dose of B7.1 or B7.1+TAP1 transfected cells dramatically protected mice from tumor challenge (Fig. 2C left), we determined if immunization of mice with γ-irradiated CMT.64 cells infected with VV-B7.1+VV-CR19 (wild-type virus) or VV-B7.1+VV-TAP1 have protection similar to mice immunized with gene-transfected tumor cells. We first analyzed B7.1 and TAP1 expression in CMT.64 cells infected overnight with VV-B7.1+VV-CR19 or VV-B7.1+VV-TAP1. We observed that B7.1 and TAP1 were highly expressed in the cells infected with relevant VVs (Fig. 5A).

Wild-type VV infection may inhibit antigen presentation in dendritic cells [20] and decrease infected cell viability. To avoid these effects, we used a recombinant VV-GFP to substitute wild-type VV-CR19 for viral-based cell infection and immunization. Mice were immunized i.p. with CMT.64 cells (5×10^6^ cells/mouse) infected with VV-GFP+VV-GFP, VV-B7.1+VV-GFP, or VV-B7.1+VV-TAP1, followed by challenge with live CMT.64 cells and survival rates were determined. Results are shown in Table 1. Mice immunized with VV-B7.1+VV-TAP1 infected CMT.64 cells showed a significantly increased survival rate, compared with mice immunized with the cells infected with VV-GFP+VV-GFP (control mice; P<0.05), while no difference was observed between control mice and mice immunized with the cells infected with VV-B7.1+VV-GFP (P>0.05). These results indicate that for immunization with a virally infected cell system, co-expression of B7.1 and TAP1 in cells is necessary to augment protective anti-tumor immunity.

**Table 1:** Increased immune protection of mice from TAP-negative tumor challenge by VV-B7.1+VV-TAP1-infected CMT.64 cell immunization.

CMT.64 cells infected with	Time (Days) 50% mice survival	No. of Mouse Survival at day 120 #	P value*
VV-GFP+VV-GFP	32	1/10	
VV-B7.1+VV-GFP	36	0/10	>0.05
VV-B7.1+VV-TAP1	79	4/10	<0.05

C57BL/6 mice (each group, n = 10) were immunized i.p with CMT.64 cells (5×10^6^ cells/mouse) infected overnight with VV-GFP+VV-GFP, VV-B7.1+VV-GFP, or VV-B7.1+VV-TAP1 at MOI of 3 for each VV. After a 20-day immunization, mice were challenged i.p. with live CMT.64 tumor cells (2.5×10^5^ cells/mouse), and the time of morbidity was recorded.

# Experiments ended at day 120 of CMT.64 cell challenge.

* Indicated that the tested mouse groups compared with a control group (immunized with VV-GFP+VV-GFP-infected CMT.64 cells) for statistical analysis (ANOVA).

Since immunization with cells expressing B7.1 alone showed different protective T-cell mediated immune responses between a virally infected cell system and a gene-transfected cell system at a dose of 5×10^6^ cells per mouse immunization, we speculated that virally derived antigens affect presentation of TAP-independent intrinsic tumor antigens, thereby decreasing the capacity of tumor cells for T-cell priming. To evaluate this possibility, standard ^51^Cr-release assays were conducted using cytolytic T cells generated by immunization with γ-irradiated CMT.B7.1/p cells (5×10^6^ cells/mouse). CMT.64 cells infected with VV-B7.1+VV-TAP1, VV-B7.1+VV-GFP or VV-GFP+VV-GFP overnight were used as targets and VV-uninfected CMT.64, CMT.B7.1/p and CMT.TAP1/B7.1 cells were used as controls. As shown in Fig. 5B, recognition of the virally infected cells by T cells was significantly reduced, compared to relevant controls.

Taken together, we conclude that effective vaccination with virally infected TAP-negative cells requires both B7.1 and TAP1 co-expression in the tumor cells.

## Discussion

This study demonstrates that B7.1 and TAP1 co-expression in TAP-negative CMT.64 cells renders tumor cells potent immunogens able to effectively induce a CD8^+^ T-cell mediated immune response against TAP-negative tumors. Induced CD8^+^ T cells likely recognize TAP-independent antigens presented by TAP-deficient tumor cells. Three important factors, TAP1, B7.1 and the amount of antigen(s) appear to contribute to CMT.64 tumor cell priming of T cells.

Our results indicate that with a low dose of γ-irradiated cell immunization, the TAP1 and B7.1 co-expressing CMT.TAP1/B7.1 cells generated an antitumor immune response greater than that induced by the TAP-negative and B7.1 expressing CMT.B7.1/p cells (Fig. 2C right). This indicates that TAP1 expression plays a critical role in enhancing T-cell priming which suggests that TAP1 expression may facilitate efficient presentation of TAP-independent tumor antigens. The possibility of efficient presentation of the antigen(s) is supported by Fig. 5B (lanes b and c) which shows that CMT.B7.1/p-induced T cells killed CMT.TAP1/B7.1 targets by better than CMT.B7.1/p targets. A possible mechanism of increased antigen presentation by TAP1 is that its expression ‘supports’ presentation of some antigens (other than ER-lumen-derived antigens which can be presented by both TAP-negative and TAP1-postive cells) that are expressed on the cell surface for T-cell recognition and/or T-cell priming. This possibility is supported by evidence that a vesicular stomatitis virus nucleocapsid protein derived epitope VSV-Np_52-59_ which is generated in the cytoplasm can be presented by TAP1 expressing CMT.64 cells more efficiently than TAP-negative CMT.64 cells [14]. Our findings highlight the multiplicity of immune responses against tumors, which are in-built into the cellular immune system. The significance of these findings should be studied further, especially for identification of TAP-independent epitopes that are well-presented and for characteristics of epitope specific T cells.

B7.1 expression in tumor cells may play a role in decreasing the threshold for antigen-specific T-cell priming [21], [22], [23]. This possibility is supported by our observation that T-cell mediated anti-CMT.64 tumor immunity can be augmented by B7.1 expressing tumor cells compared with B7.1 negative tumor cells (Fig. 2C left). Mechanisms involved in priming of T cells against CMT.64 tumors by B7.1 expressing TAP-deficient cells may be preferentially through tumor direct priming rather than dendritic cell (DC) cross-priming. This explains why immunization with B7.1 expressing CMT.64 cells induced a high level of anti-CMT.64-tumor immunity while immunization with B7.1-negative CMT.64 cells provided significantly less immunity. Cross-priming requires professional antigen presenting DCs to capture antigenic proteins from apoptotic cells and to process and present the generated epitopes for T-cell priming in a TAP-dependent manner [24]. Thus, TAP-independent epitopes which are expressed on CMT.64 cells may not be presented on the DC's surface for T-cell priming, because these epitopes similar to the peptides presented on TAP-deficient RMA-S cells [25] generally display low ability for MHC-I binding, and therefore cannot compete with TAP-dependent epitopes for MHC-I binding and DC's surface expression.

The amount of antigen(s) used for immunization directly affects the strength of generated antitumor immunity as confirmed by our results regarding dose-dependent immunization with γ-irradiated cells (Fig. 2C left and right). With a high dose cell immunization, highly protective immunities were generated by both CMT.TAP1/B7.1 and CMT.B7.1/p cells. This suggests that both γ-irradiated cell lines supplied efficient amounts of TAP-independent antigens for T-cell induction. With a low dose immunization, TAP1-positive CMT.TAP1/B7.1 cells stimulated the immune system to generate protective immunity better than TAP-negative CMT.B7.1/p cells. This suggests that TAP1 and B7.1 co-expressing CMT.TAP1/B7.1 cells provide TAP-independent antigens for T-cell priming more than that provided by B7.1 expressing CMT.B7.1/p cells.

A recent report illustrated that immunization with B7.1-transfected TAP1-expressing RMA-S cells elicited T-cell based protective immunity against RMA-S cells [13]. Our results further show that immunization with either TAP1-expressing or TAP-negative tumor cells that express B7.1 can elicit an effective T-cell mediated immune response against TAP-negative tumor challenge. In such protective immunity, CD8^+^ T cells played a major role in the response to TAP-negative tumor cells, while CD4^+^ T cells had a significant role as well. Since CMT.64 cells do not express MHC class II molecules, a major function of CD4^+^ T cells may be to support CD8^+^ T-cell responses to tumor cells [26], [27], [28], [29], [30], [31], [32] or through a cytokine release mechanism to accumulate and activate effector cells [33]. NK activity may not play a major role in the immune response against CMT.64 tumor cells, as we did not observe high protective immunity in cell-immunized experiments in which the CD8^+^ T-cell population only was depleted by antibody, whereas the NK population should have been present (Fig. 3B left).

We have confirmed that TAP-deficient CMT.64 transfectants are able to present TAP-independent Lass5 epitope and that with B7.1 expression, such cells can elicit Lass5-specific T cells. These results provide direct evidence for an immune response against a TAP-independent antigen. Although tumor cells can present the Lass5 epitope and elicit T cells, we believe that generation of tumor-rejective T-cell mediated immunity may require another TAP-independent antigen or multiple antigens for T-cell priming because the Lass5 epitope is presented at a low level in CMT.64 cells. This is suggested by the prolonged time requirement for killing the cells and by the relatively low number of Lass5 antigen specific T cells.

In summary, B7.1 and TAP1 co-expression in TAP-negative tumor cells provides two major advantages for induction of a T-cell mediated antitumor immune response. TAP1 expression affords tumor cells the ability to efficiently present TAP-independent antigen(s) while B7.1 expression in tumor cells decreases the threshold for priming of TAP-independent antigen specific T cells. Thus, expression of both genes in tumor cells can facilitate the induction phase of the T-cell based immune response. Our findings may encourage the potential use of both genes for cancer immunotherapy in the future.

## Materials and Methods

### Animals

C57BL/6 (H-2^b^) and nude mouse (H-2^b^) strains were obtained from the NCI and housed in the animal facility at LSU Health Sciences Center. All mice used for the experiments were 6–10 wk old females and were maintained and treated in accordance with the guidelines of the Animal Use Committee at LSU Health Sciences Center.

### Vectors

Wild-type vaccinia virus (VV) VV-CR19 [34] and recombinant VV carrying an entire TAP1 gene (VV-TAP1), or influenza A virus nucleoprotein minigene NP_366–374_ (VV-NP_366–374_) were kindly provided by Dr. Yewdell, J (Laboratory of Viral Diseases, National Institute of Allergy and Infectious Diseases, Bethesda, MD). VV carrying a GFP gene (VV-GFP) was kindly provided by Dr. Kirkegaard, K (Microbiology and Immunology, Stanford University, School of Medicine, Stanford, CA). VV carrying a B7.1 gene [35] (VV-B7.1) was kindly provided by Dr. Schlom, J (Laboratory of Tumor Immunology and Biology, Center for Cancer Research, Bethesda, MD). Mouse TAP1 and TAP2 cDNA was inserted into the vectors pcDNA3.1/His and pUB6/V5-His (Invitrogen Corporation, Carlsbad, CA) as described previously [36]. A B7.1-vector [37] was provided by Dr. Li, S (Louisiana State University, Baton Rouge, LA). B7.1 cDNA was isolated and inserted into a pUB6/V5-His vector (Invitrogen Corporation).

### Cell lines and cell culture

The mouse lung carcinoma cell line CMT.64-7 designated as CMT.64 [38] was kindly provided by Dr. Gunnel Hallden (Imperial College of Science, London, UK). CMT.64/pp, CMT.B7.1/p, CMT.TAP1/p, CMT.TAP1/B7.1 and CMT.TAP1,2 cl.21 (clone 21) cells were generated by co-transfection of two empty vectors (pcDNA3.1/His and pUB6/V5-His), B7.1+pcDNA3.1/His Vectors, TAP1+pUB6/V5-His, B7.1+TAP1 vectors and TAP1+TAP2 vectors respectively. Transfectants were maintained in DMEM medium containing 10% FBS, 1000 µg/ml neomycin and 20 µg/ml blasticidin selection medium. CMT.64 cells were cultured in DMEM medium while RMA and RMA-S cells were cultured in RPMI 1640 medium. RMA-S transfected with B7.1 (designated as RMA-S/B7.1) cells were cultured with RPMI 1640 supplemented with 20 µg/ml blasticidin selection medium.

### Detection of Lass5 expression by RT-PCR

Lass5 protein is an endoplasmic reticulum (ER)-membrane spanning protein [13]. The Lass5 gene transcribes two mRNA splice variants that include or exclude exon 9a and these two variants are termed Lass5 long and short transcripts respectively [13]. The long transcript encodes a Lass5 protein containing a D^b^-restricted epitope MCLRMTAVM at the protein C-terminal end [13]. Lass5 short and long transcripts were detected by RT-PCR using the shared upstream primer 5′-GCAGACCCCTTACTGGAAGCTGCC-3′ and the specific downstream primers 5′-CGGTCATCCTTAGACACATGCAAAGG-3′ (for long transcript) or 5′-CTGCGGTCATCCTTAGACACCTTTCC-3′ (for short transcript).

### MHC-I and B7.1 expression

Surface MHC-I and B7.1 expression was detected by a FACScan analyzer (Becton Dickinson, Mountain View, CA) using direct and indirect immunofluorescence. For MHC-I expression, cells were stained with an K^b^-specific Y-3 primary monoclonal antibody (mAb) (ATCC, Manassas, VA) or an D^b^-specific 28-14-8S primary mAb (ATCC) followed by staining with a FITC-conjugated goat anti-mouse IgG secondary antibody (Ab) (Jackson ImmunoResearch, West Grove, PA). CMT.64 cells stained with a primary mAb 15-5-5S (against H-2D^k^, ATCC) followed by staining with a FITC-conjugated goat anti-mouse IgG secondary antibody (Ab) were used as a negative control. For B7.1 expression, a FITC-conjugated anti-mouse CD80 mAb (clone 16–10A1 purchased from BioLegend) was used.

### Detection of TAP

TAP protein was determined by Western blot using a goat anti-mouse TAP1 polyclonal Ab (for TAP1, Santa Cruz Biotechnology, Santa Cruz, CA) or mouse antiserum (for TAP2, generated in our laboratory [36]). Western blot protocol was described previously [36]. For a loading control, the levels of expression of the enzyme glyceraldehyde-3-phosphate dehydrogenase (GAPDH) protein was detected in each sample as described previously [19], [39].

### Generation of cytolytic T lymphocytes (CTL) and cytotoxicity tests

Antigen specific CTLs were generated by immunization of C57BL/6 mice with γ-irradiated RMA-S/B7.1 cells (1×10^7^ cells/mouse) pulsed with 5 µM Lass5 peptide (for D^b^-restricted Lass5-CTLs) or pulsed with influenza NP_366–374_ (ASNENMDTM) peptide (for D^b^-restricted NP_366–374_-CTLs). Tumor-antigen specific CTLs were generated by immunization of C57BL/6 mice with γ-irradiated CMT.B7.1/p cells (5×10^6^ cells/mouse). After a 9-day immunization, the splenocytes were cultured with peptide or γ-irradiated and mitomycin-c-treated (30 µg/ml) cells for 5 days and then used as effectors. For Lass5 antigen, the immunized splenocytes were re-stimulated with Lass5-peptide pulsed RMA-S/B7.1 cells (1×10^7^ cells/immunized-spleen) and 12–16 h ^51^Cr-release assays were conducted. For tumor-antigen specific CTLs, the immunized splenocytes were re-stimulated with CMT.B7.1/p cells (1×10^7^ cells/immunized-spleen) and 4-h standard ^51^Cr-release assays were conducted. For NP_366–374_-CTLs, the immunized splenocytes were re-stimulated with either (1×10^7^ cells/immunized-spleen) NP_366–374_-peptide pulsed RMA-S/B7.1 cells (Figure 4B left) or with 5 µM NP_366–374_ peptide (Figure 4B right). 12–16 h (Figure 4B left) or 4-h (Figure 4B right) ^51^Cr-release assays were conducted. The ^51^Cr-labeled targets are described in each figure or figure legend. Three experiments were performed for each antigen or tumor cell type. One representative of three experiments (Fig. 4) or the mean value (mean±SEM) from three experiments (Fig. 5B) is shown.

### ELISPOT analysis of TAP-independent Lass5 antigen-specific INF-γ secreting splenocytes

C57BL/6 mice were immunized i.p with γ-irradiated tumor cells (5×10^6^ tumor cells/mouse) two times (with a 12-day interval). Nine days after the last immunization, splenocytes were isolated and cultured *in vitro* in RPMI 1640 complete medium with or without 5 µM Lass5 peptide MCLRMTAVM for 14 hours. The frequency of Lass5 specific IFN-γ secreting cells was determined using an ELISPOT assay (R&D Systems, Minneapolis, MN) according to the manufacturer's instructions.

### Detection of tumorigenicity and Tumor challenge experiments

For detection of tumorigenicity, C57BL/6 mice or nude mice (each group, n = 10) were injected i.p. with 5×10^4^ tumor cells (in 500 µl PBS), and the time of morbidity was recorded. For tumor challenge experiments, C57BL/6 mice (each group, n = 10) were immunized i.p. with γ-irradiated tumor cells, and after a 20-day immunization the mice were challenged i.p. with live CMT.64 tumor cells (2.5×10^5^ cells/mouse), and the time of morbidity was recorded. Preparation of γ-irradiated tumor cells for immunization followed the procedures as shown below. CMT.64 transfectants were γ-irradiated at a 10000-rads irradiation dosage (for Fig. 2C and Fig. 3B), or CMT.64 cells were infected with VVs overnight (16 hours) followed by γ-irradiation (10000-rads) and treatment with mitomycin-c (30 µg/ml) for 2-hours. After extensive wash with PBS, the cells (in 500 µl PBS per mouse) were inoculated i.p into C57BL/6 mice. The number of cells used for immunization is noted in each figure legend and table 1. Statistics for mouse survival were obtained using the Kaplan–Meier log rank survival test, and differences were considered significant at P<0.05. For in vivo experiments, % survival was displayed in each figure, instead of the time of morbidity. This is because the morbidity to death for the mice inoculated i.p. with CMT.64 tumor cells was about 3–4 days. Therefore, the time of morbidity can refer to the survival time extending 3–4 days.

### Depletion of T cell sub-population in C57BL/6 mice using relevant antibodies

Depletions of effectors *in vivo* were started 1 week before vaccination. mAb GK1.5 or mAb 2.43 (ATCC, Manassas, VA) was used for CD4 and CD8 depletions respectively [40]. mAb purification and animal injection followed from a report [40] with slight modification. Tissue cultured mAbs were purified using the ammonium sulfate method and the purified mAbs had titers at about 1∶2000 by staining of splenocytes detected by FACS. C57BL/6 mice were injected i.p with 0.1 ml/mouse mAb every other day for the first week and once per week afterward. Depletion of lymphocyte subsets was assessed in blood on and after the day of tumor cell vaccination and live tumor challenge by using FITC-conjugated anti-mouse CD8a (5H1-1) or FITC-conjugated anti-mouse CD4 (RM4-4) together with PE/Cy5-conjugated anti-mouse CD3 (145-2C11) (all from BioLegend). After depletion of CD8^+^ or CD4^+^ T cell subsets, the mice were immunized with γ-irradiated tumor cells (5×10^6^ cells per mouse), and after a 20-day immunization the mice were challenged with live CMT.64 tumor cells (2.5×10^5^ cells per mouse). The time of morbidity was recorded.
